# Urinary Biomarkers Improve the Diagnosis of Intrinsic Acute Kidney Injury in Coronary Care Units

**DOI:** 10.1097/MD.0000000000001703

**Published:** 2015-10-09

**Authors:** Chih-Hsiang Chang, Chia-Hung Yang, Huang-Yu Yang, Tien-Hsing Chen, Chan-Yu Lin, Su-Wei Chang, Yi-Ting Chen, Cheng-Chieh Hung, Ji-Tseng Fang, Chih-Wei Yang, Yung-Chang Chen

**Affiliations:** From the Department of Nephrology, Kidney Research Center, Taipei, Taiwan (C-HC, H-YY, C-YL, C-CH, J-TF, C-WY, Y-CC); Department of Cardiology, Chang Gung Memorial Hospital, Taipei, Taiwan (C-HY, T-HC); Clinical Informatics and Medical Statistics Research Center, Taipei, Taiwan (S-WC); Department of Biomedical Sciences, Chang Gung University, Taoyuan, Taiwan (Y-TC); and College of Medicine, Chang Gung University, Taoyuan, Taiwan (C-HC, H-YY, C-YL, C-CH, J-TF, C-WY, Y-CC)

## Abstract

Acute kidney injury (AKI) is associated with increased morbidity and mortality and is frequently encountered in coronary care units (CCUs). Its clinical presentation differs considerably from that of prerenal or intrinsic AKI. We used the biomarkers calprotectin and neutrophil gelatinase-associated lipocalin (NGAL) and compared their utility in predicting and differentiating intrinsic AKI.

This was a prospective observational study conducted in a CCU of a tertiary care university hospital. Patients who exhibited any comorbidity and a kidney stressor were enrolled. Urinary samples of the enrolled patients collected between September 2012 and August 2013 were tested for calprotectin and NGAL. The definition of AKI was based on Kidney Disease Improving Global Outcomes classification. All prospective demographic, clinical, and laboratory data were evaluated as predictors of AKI.

A total of 147 adult patients with a mean age of 67 years were investigated. AKI was diagnosed in 71 (50.3%) patients, whereas intrinsic AKI was diagnosed in 43 (60.5%) of them. Multivariate logistic regression analysis revealed urinary calprotectin and serum albumin as independent risk factors for intrinsic AKI. For predicting intrinsic AKI, both urinary NGAL and calprotectin displayed excellent areas under the receiver operating characteristic curve (AUROC) (0.918 and 0.946, respectively). A combination of these markers revealed an AUROC of 0.946.

Our result revealed that calprotectin and NGAL had considerable discriminative powers for predicting intrinsic AKI in CCU patients. Accordingly, careful inspection for medication, choice of therapy, and early intervention in patients exhibiting increased biomarker levels might improve the outcomes of kidney injury.

## Abstract 2

Supplemental Digital Content is available in the text

## INTRODUCTION

Acute kidney injury (AKI) is a common complication responsible for increased medical expenditure and poor outcome in hospital settings.^1,2^ Its incidence varies from 28% to 75% based on etiologies and has increased in the past decade.^3–7^ In addition, AKI developing after admission to a coronary care unit (CCU) is not only associated with 10-fold increased mortality but also long-term complications.^8,9^ Even minor alterations in serum creatinine (SCr) levels (>0.25 mg/dL) following angiogram are associated with increased mortality.^10^ In 2007, the Acute Kidney Injury Network (AKIN) group proposed modified standard criteria called RIFLE: Risk of renal failure, Injury to the kidney, Failure of kidney function, Loss of kidney function, and End-stage renal failure. Based on the RIFLE criteria, the AKIN group enrolled patients who exhibited an increase in SCr levels to 0.3 mg/dL within 48 hours. Although this change increased the sensitivity of AKI detection, several studies have assessed and suggested that these criteria enrolled patients with prerenal AKI.^11,12^ Patients admitted to CCUs typically exhibit complex syndromes with numerous pathways that affect renal function. Especially, AKI typically develops following acute myocardial infraction (AMI), congestive heart failure (CHF), arrhythmia, sepsis, and contrast medium injection, which also cause prerenal AKI. In addition, medical interventions such as fluid restriction and administration of diuretics, angiotensin-converting enzyme inhibitors, and aldosterone receptor blockers increase the risk of prerenal azotemia and kidney injury. By definition, renal dysfunction that recovers within 72 hours after injury is termed prerenal AKI.^13^ Reportedly, prerenal azotemia occurs in 32.1% of AKI cases in hospital settings and is associated with increased mortality.^13^ Two other studies have revealed that patients in whom AKI persists for>3 days exhibit higher long-term mortality than do those in whom AKI resolved within 3 days.^14,15^ Despite the lack of any previous investigations, we believe that the prevalence of prerenal AKI is much higher in CCU settings as a result of cardiac dysfunction and diuretics usage. In contrast, intrinsic AKI is caused by prolonged or severe kidney injury.^16^ At the cellular biology level, an intrinsic injury leads to apoptosis or necrosis of the renal tubule cells that fail to adapt to the stress. Traditionally, fractional excretion of sodium (FE_Na_) and urea (FE_Urea_) are commonly used parameters to discriminate prerenal from intrinsic AKI. However, certain drawbacks have been disclosed in previous studies. For example, FE_Na_ is unreliable in patients who are older or those with heart failure or diuretic use. FE_Urea_ is affected by protein and fluid intake, and it also lacks specificity.^17–19^ Currently, novel biomarkers are developed to identify patients with renal tubular injury, which could be helpful for early prediction of the clinical course of the disease and help doctors in making appropriate clinical decisions.^20,21^ However, a study reported mildly increased neutrophil gelatinase-associated lipocalin (NGAL) in patients who developed prerenal AKI after cardiac surgery.^22^ Moreover, studies have reported calprotectin as a favorable differential marker for prerenal AKI.^23,24^ Thus, the purpose of this research was to exam whether NGAL and calprotectin can distinguish intrinsic from prerenal disease without dedicating whole paragraphs on AKI but focus on cardio-renal syndrome. To the best of our knowledge, this is the first investigation that combined risk-evaluation strategy and biomarkers to discriminate intrinsic AKI in a CCU setting. The findings of this study may guide practitioners in identifying and treating patients at risk of AKI.

## MATERIALS AND METHODS

### Study Design, Patient Information, and Data Collection

This cross-sectional study was performed in the CCU at a tertiary care referral center in Taiwan between September 2012 and August 2013. The study protocol was approved by the local Institutional Review Board. Patients who exhibited any of the comorbidities of AKI and any of the kidney stressors were enrolled in this investigation. The following comorbidities were considered as risk factors for AKI: age >65 years, diabetes mellitus, CHF (functional class III or IV), and chronic kidney disease (CKD, defined as estimated glomerular filtration rate ≤60 mL/min).^25^ In addition, the following conditions when observed during the period from before admission were considered as kidney stressors: AMI (defined according to the 2007 Expert Consensus Document of Circulation, European Heart Journal), shock (systolic pressure ≤90 mm Hg, or use of any inotropic agent to maintain blood pressure or cardiac output), arrhythmia (ventricular fibrillation, ventricular flutter, or third-degree atrioventricular block), sepsis (defined according to the American College of Chest Physicians/Society of Critical Care Medicine [ACCP/SCCM] Consensus Conference), renal toxin exposure (contrast medium, nonsteroidal anti-inflammatory drug, and aminoglycoside), and mechanical ventilator support. Patients who were receiving dialysis, ages <18 years, or reported prior organ transplantation were excluded. To ensure early detection, only those patients who were admitted to CCU within 72 hours from the emergency department were enrolled. Because calprotectin was considered as a confounding factor, patients with urinary tract infections or gross hematuria after indwelling urinary catheter were excluded based on a previous study.^24^ According the study, the median level of calprotectin for the healthy control and intrinsic AKI was 45 ng/mL (19–139 ng/mL) and 1692 ng/mL (765–4735 ng/mL), respectively.^24^ Given the type I error of 0.05 and power of 0.90, resulting a minimum sample size of 72 urinary calprotectin data to detect an intrinsic AKI. The determination of sample size was performed using nonparametric Mann–Whitney *U* test procedure of NCSS 2007 (Number Cruncher Statistical System, Limited Liability Company, Kaysville, Utah).

To determine the predictive value of biomarkers for intrinsic AKI, the primary outcome was the development of AKI within 7 days after admission. Based on the Kidney Disease Improving Global Outcomes (KDIGO) Clinical Practice Guidelines for Acute Kidney Injury, AKI was confirmed under any of the following conditions: SCr levels ≥0.3 mg/dL within 48 hours or ≥1.5 times increase in SCr levels from baseline within 7 days.^26,27^ In addition, the severity of AKI was staged according to the KDIGO guideline. Definitions of prerenal AKI and intrinsic AKI were modified based on a prior study conducted by the Westhoff group (Table 1).^24^ To assess the prognostic utility of biomarkers, 6-month mortality was considered as the secondary outcome. After hospital discharge, 6-month follow-up examinations were performed by reviewing the follow-up records or using telephone interviews as needed.

**TABLE 1:**
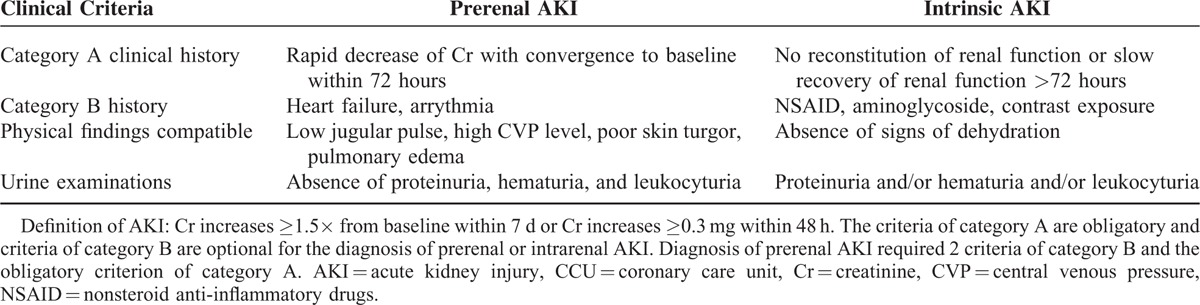
Diagnostic Criteria for Prerenal AKI and Intrinsic AKI in CCUs

The following data were collected prospectively: demographic characteristics, primary diagnosis, routine biochemistry tests, and treatment outcomes. Overall, 151 patients provided informed consent, but 4 of these were excluded from analysis because repeated kidney injuries were observed after sample collection. A total of 147 patients were enrolled in this study.

### Sampling and Quantifying Urinary Neutrophil Gelatinase-Associated Lipocalin and Calprotectin

Urinary samples were collected in sterile nonheparinized tubes immediately after admission and then centrifuged at 5000×*g* for 30 minutes at 4°C to remove cells and debris. The clarified supernatants were stored at −80°C for 6 months before measurement. Urinary neutrophil NGAL and calprotectin levels were measured in duplicate by using commercially available enzyme-linked immunosorbent assay (ELISA) kits according to the manufacturer's instructions (R&D Systems, DLCN20, McKinley Place NE Minneapolis; MPLS, USA and Phi Cal^®^ Calprotectin, K 6935; and Immundiagnostik AG, Bensheim, Germany). The intra-assay coefficients of variability for urine NGAL and urine calprotectin were 2.29% and 0.9%, respectively. The inter-assay coefficients of variability for NGAL and urine calprotectin were 9.06% and 11.28%, respectively (n = 4).

### Statistical Analysis

Continuous variables were shown as the mean and standard error (SE). The Kolmogorov–Smirnov test was used to determine the normal distribution of each variable. The continuous variables of the non-AKI, prerenal AKI, and intrinsic AKI groups were analyzed using repeated-measures analysis of variance (ANOVA) by the Tukey's honestly significant difference test for post hoc analysis. The categorical data were analyzed using χ^2^ test. The predictive parameters for intrinsic AKI were assessed using univariate analysis, and the statistically significant variables were included in multivariate analysis by using a multiple logistic regression model based on backward elimination of data.

The Hosmer–Lemeshow goodness-of-fit test was used for calibration when evaluating the number of observed and predicted intrinsic AKI cases in the risk groups for the entire range of probabilities. Discrimination was assessed using the area under the receiver operating characteristic curve (AUROC). The AUROC analysis calculated cut-off values, sensitivity, specificity, and overall accuracy. Subsequently, cut-off points were calculated by acquiring the optimal Youden index. The Youden index has minimum and maximum values of −1 and +1, respectively, and a value of +1 is considered the optimal value for an algorithm. Cumulative survival curves were assessed using the Kaplan–Meier approach. To determine the combined discriminative ability of biomarkers, the multiple logistic regression model was applied with both NGAL and calprotectin as independent variables. *P* value of <0.05 was considered statistically significant.

## RESULTS

### Study Population Characteristics

**FIGURE 1:**
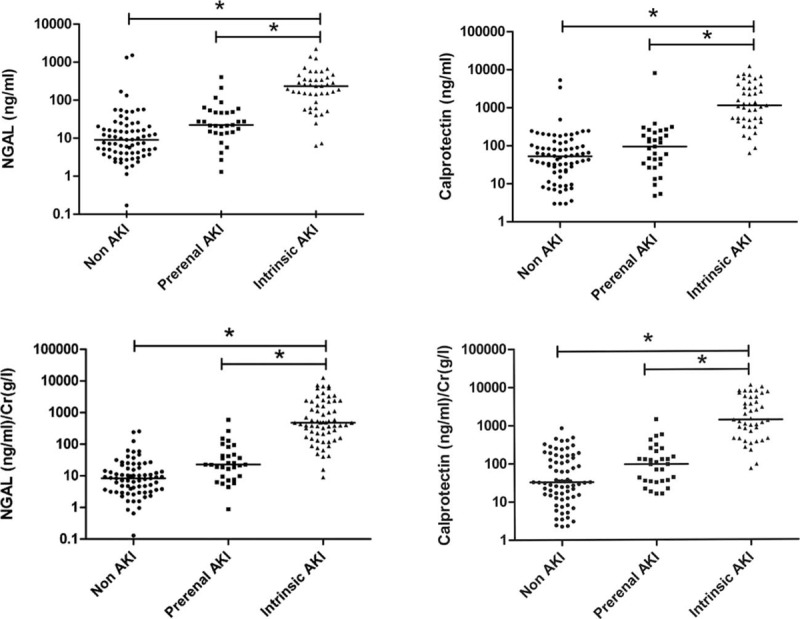
Individual measurement results of urinary NGAL and calprotectin levels of intrinsic acute kidney injury (AKI), prerenal AKI, and non-AKI of the complete study population. The data are presented as scatter plots (logarithmic scale; medians are indicated by horizontal lines). Both concentrations of NGAL and calprotectin in intrinsic AKI were significant higher than prerenal and non-AKI. NGAL = neutrophil gelatinase-associated lipocalin.

Overall, 147 adult patients (100 men and 47 women), with a mean age of 67 years were investigated. AKI was diagnosed in 71 (50.3%) patients, of which, 43 (60.5%) were diagnosed with intrinsic AKI. The patient characteristics included age, sex, hematological parameters, and biomarker levels, as listed in Table 2. Hypertension and coronary arterial disease were recorded in 69% of patients during recruitment. The mean urinary NGAL levels were 56.1 ± 28.5, 49.4 ± 14.4, and 329.3 ± 63.6 ng/mL in the non-AKI, prerenal, and intrinsic AKI groups, respectively (*P* < 0.001); moreover, the mean urinary calprotectin levels were 195.8 ± 89.0, 385.2 ± 271.7, and 2404.5 ± 417.9 ng/mL, respectively (*P* < 0.001) (Figure 1). The mean urinary NGAL levels were 56.1 ± 28.5, 49.4 ± 14.4, and 329.3 ± 63.6 ng/mL in the non-AKI, prerenal, and intrinsic AKI groups, respectively (*P* < 0.001); moreover, the mean urinary calprotectin levels were 195.8 ± 89.0, 385.2 ± 271.7, and 2404.5 ± 417.9 ng/mL, respectively (*P* < 0.001). For large variability observed within the 3 groups, the NGAL and calprotectin was adjusted to urine Cr. The mean urinary NGAL/Cr levels were 56.1 ± 28.5, 49.4 ± 14.4, and 329.3 ± 63.6 ng/mL in the non-AKI, prerenal, and intrinsic AKI groups, respectively (*P* < 0.001); and, the mean urinary calprotectin/Cr levels were 195.8 ± 89.0, 385.2 ± 271.7, and 2404.5 ± 417.9 ng/mL, respectively (*P* < 0.001). Nine intrinsic AKI group patients had received hemodialysis. Overall, 14 (9.5%) patients died before the 6-month follow-up. Table 2 summarizes the patient characteristics according to the different AKI categories. The intrinsic AKI group patients were significantly older and exhibited significantly higher SCr levels, WBC counts, and B-type natriuretic peptide (BNP), NGAL, and calprotectin levels than did the non-AKI group patients. In addition, the intrinsic AKI group patients exhibited lower coma scale scores and albumin, hemoglobin, and serum sodium levels than did the non-AKI group patients. However, the prerenal and intrinsic AKI groups revealed significant differences in only the hemoglobin, NGAL, and calprotectin levels. Table 3 lists the characteristics evaluated for risk-factor analysis among all groups. The intrinsic AKI group patients exhibited significantly higher incidence of severe heart failure, CKD, and sepsis. The ratio of AMI and renal toxin exposure was significantly lower in the intrinsic AKI group. In addition, significant differences were observed in the cumulative survival rates (*P* < 0.05) between the non-AKI and the prerenal and intrinsic AKI groups at the 6-month follow-up (Appendix 2, http://links.lww.com/MD/A447). Unfavorable outcome was also noted in the patients whose NGAL exceeded 39.0 ng/mL or calprotectin exceeded 314.6 ng/mL, respectively (Appendices 3 and 4, http://links.lww.com/MD/A447).

**TABLE 2:**
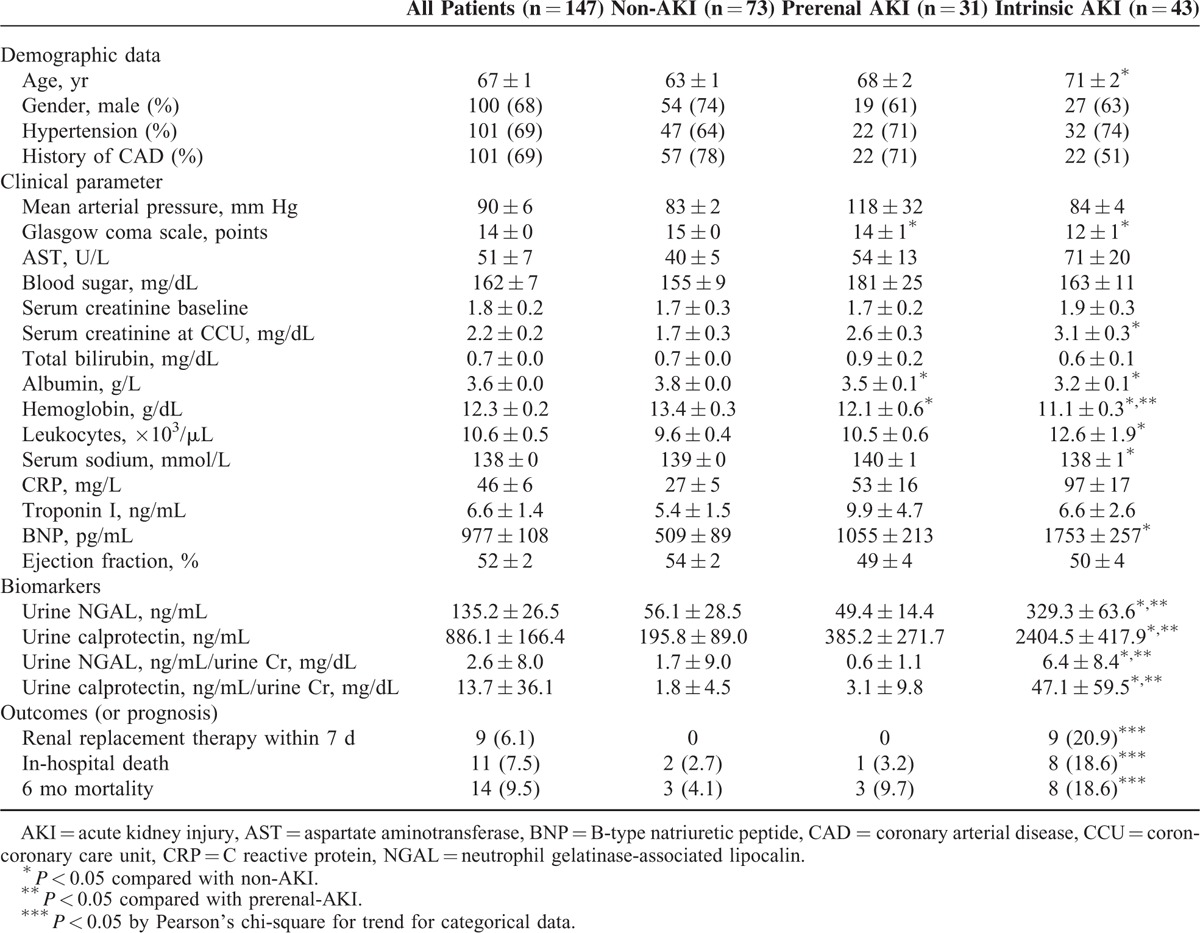
Demographic Data and Clinical Characteristics on Admission of Non-AKI, Prerenal AKI, and Intrinsic AKI Group Patients

**TABLE 3:**
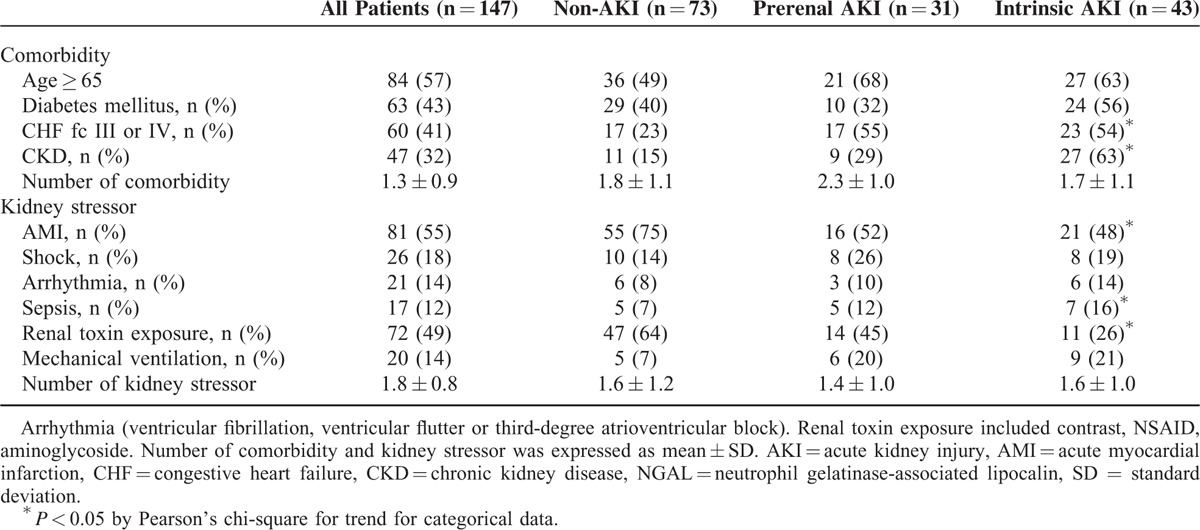
Risk Factor Analysis for Intrinsic AKI

### Urinary NGAL and Calprotectin in Intrinsic AKI

**FIGURE 2:**
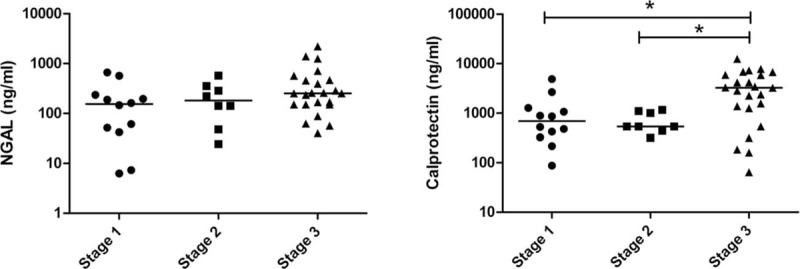
Different expression levels of urinary NGAL and calprotectin according to differ severity of AKI. Only concentrations of calprotectin in stage 3 AKI show the differences compared with AKI stage 1 and stage 2. AKI = acute kidney injury, NGAL = neutrophil gelatinase-associated lipocalin.

Although age, coma scale scores, severe CHF, AMI, and SCr, albumin, hemoglobin, BNP, CKD, urinary NGAL, and calprotectin levels were associated with AKI according to the univariate analysis, only SCr, hemoglobin, and calprotectin levels were independently associated with intrinsic AKI according to the multivariate analysis (Table 4). For a 1-U (1 pg/mL) increase in the calprotectin levels, an approximately 0.1% increase was expected in the risk of that particular event (95% confidence interval [CI] = 1.000–1.001, *P* < 0.001). The levels of both biomarkers were compared according to AKI staging. No significant differences were observed in the NGAL levels across the various aforementioned categories. Concurrently, the urinary calprotectin levels at stage 3 AKI were significantly higher than those at the other stages (Figure 2).

**TABLE 4:**
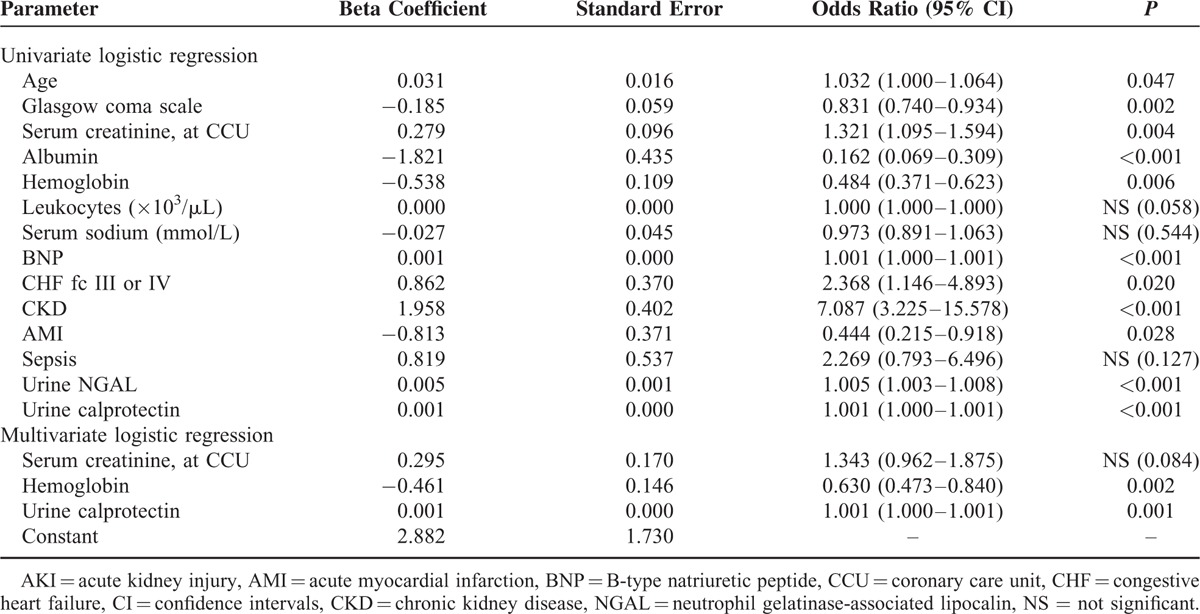
Logistic Regression Analysis for Intrinsic AKI Based on Baseline Prognostic Factors on CCU Admission

### Discrimination Power of Urinary NGAL and Calprotectin in Predicting Intrinsic AKI

The accuracy of the urinary biomarkers NGAL and calprotectin in the detection of intrinsic AKI was assessed through ROC curve analysis (Table 5). The ROC analysis of NGAL and calprotectin revealed AUROCs of 0.918 and 0.946, respectively. The Youden index was used to determine the optimal cut-off value to differentiate prerenal and intrinsic AKI. As shown in Appendix 1, http://links.lww.com/MD/A447, NGAL exhibited a sensitivity of 93.0% and a specificity of 83.0% for a threshold value of 39.0 ng/mL, whereas urinary calprotectin exhibited a sensitivity of 88.4% and a specificity of 96% for a cut-off value of 314.6 ng/mL. The overall accuracy of NGAL and calprotectin was 83.7% and 91.2%, respectively. Compared with calprotectin alone, a combination of both markers did not improve the diagnostic accuracy (AUROC = 0.946), indicating there was no incremental value of NGAL in diagnosing intrinsic AKI when calprotectin was considered.

**TABLE 5:**
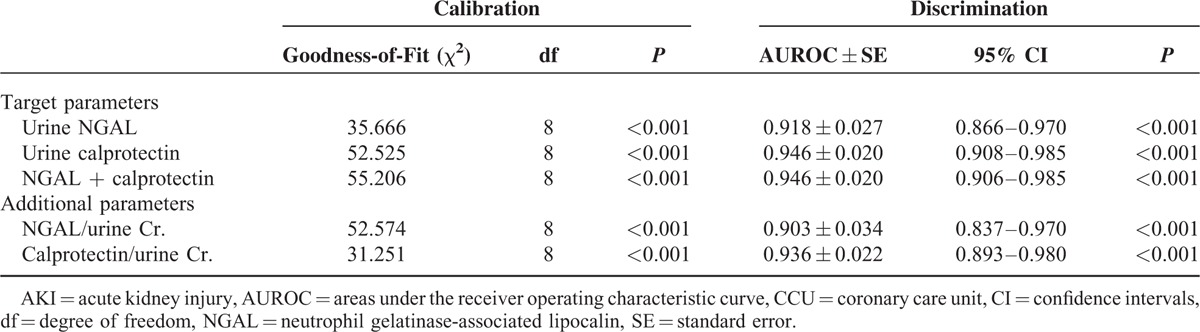
Comparison of Calibration and Discrimination of Biomarkers on the First Day of CCU Admission in Predicting Intrinsic AKI

## DISCUSSION

Patients admitted to CCUs typically exhibit complex syndromes with numerous pathways that affect renal function. Decision making for patients with AKI is challenging, particularly when the treatments administered to patients with prerenal AKI and those with intrinsic AKI are markedly different. We conducted a biomarker study integrated with a risk-evaluation strategy. Compared with the findings of our previous study, the incidence of AKI increased twofold, from 28.7% to 50.3%, which suggests increased utility of biomarkers in detecting AKI in high-risk groups.^8^ To the best of our knowledge, this is the first investigation that combined risk-evaluation strategy and biomarkers to discriminate intrinsic AKI from the other types in a CCU setting. In this study, the intrinsic AKI group patients were older and exhibited worse conditions on admission than did the non-AKI group patients. However, only serum albumin and urinary biomarkers could differentiate intrinsic AKI from prerenal AKI. Patients with AMI, whether or not undergoing primary PCI, are at high risk for AKI and for persistent renal damage.^28–30^ Moreover, several other studies have reported that the incidence of AKI following primary percutaneous coronary intervention ranged from 2% to 20.6%, and most of these studies have reported favorable renal out comes for such patients.^31–35^ Impaired cardiac function also compromises renal perfusion via arterial vasoconstriction, venous congestion and results in sodium water retention. BNP, associated with heart failure, has its prognostic role and predicts the development of AKI.^36,37^

NGAL, also known as lipocalin-2 (LCN2), is a 25-kDa protein encoded by the LCN gene, which was originally identified in neutrophils as a shuttle for iron transport to inhibit bacterial growth.^38^ In addition, NGAL expression has been identified in the kidney and liver in response to inflammation, infection, intoxication, ischemia, neoplastic formation, and AKI.^39–42^ NGAL also has antiapoptotic effects and an ability to induce tubular cell proliferation, and these constitute the possible pathways of NGAL-mediated kidney protection in AKI. In a healthy population, minute levels of NGAL are filtrated from inside the glomerulus and the luminal NGAL is reabsorbed in the proximal tubule through a megalin-dependent pathway.^43^ Immediately after AKI, NGAL is upregulated in the ascending limb of the Henle's loop, the distal tubule, and the collecting duct. Moreover, impaired proximal reabsorption during the onset of proximal tubular absorption upregulates urinary NGAL levels.^43^ Numerous studies have reported the potential clinical utility of NGAL in AKI. In our previous study, urinary NGAL revealed an AUROC of 0.796 with a cut-off value of 33 ng/mL.^8^ This finding provides further evidence that NGAL may be effective in treating AKI.

Calprotectin (S100A8/S100A9) is a 36-kDa heterodimer of 2 calcium-binding proteins identified in the cytoplasm of neutrophils and monocytes that was initially discovered as an antimicrobial protein.^44^ Calprotectin is released in the circulation or body fluids on neutrophil activation or endothelial adhesion. In addition, it is implicated in the recruitment of inflammatory cells to amplify the inflammatory cascade through innate immunity with Toll-like receptor (TLR) 4 as the damage-associated molecular pattern protein.^45,46^ Several studies have demonstrated its role in inflammatory bowel disease, cystic fibrosis, asthma, heart failure, and AMI.^47,48^ Innate immunity is activated through TL2 and TL4 present in the tubular cells that are involved in the cellular mechanism of AKI.^49–51^ Caspase-1 mediates inflammation via the activation of interleukin-1β and interleukin-18 (IL-18).^22,52^ Caspase-3 is a major mediator of both apoptotic and necrotic cell death and also involves in calprotectin-mediated apoptosis.^45^ Increases in both caspase-1 and caspase-3 have been described in is chemic injury to various organs including brain, heart, and kidney.^53,54^ These evidences support the role of inflammation on cardiorenal interaction.

In the present study, urinary calprotectin may have been released from the infiltrating leukocytes because of tubular damage or circulation. In a study on 101 patients with AKI admitted in a nephrology ward, Westhoff et al demonstrated that urinary calprotectin exhibited an AUROC of 0.97 with a cut-off value of 300 ng/mL, wherein various diseases, such as hepatorenal syndrome, caridorenal syndrome, and renal artery stenosis, were evaluated. In this study, patients with urinary tract infections also exhibited significantly increased calprotectin levels.^24^ They also conducted a second investigation on 87 patients (38 with AKI, 24 with prerenal AKI, and 25 healthy controls) and reported that urinary calprotectin exhibited an AUROC of 0.99, where as NGAL exhibited an AUROC of 0.82 with cut-off values of 440 and 52 ng/mL, respectively. Another research by Han et al^55^ reported that a combination of biomarkers might improve the accuracy of diagnosing AKI after cardiac surgery. In our study, the discriminatory powers of calprotectin, urinary NGAL, or a combination of these markers were similar (0.918 vs 0.946 vs 0.946, respectively). There were 2 possible explanations. First, urinary NGAL is immediately released after open heart surgery, whereas persistent elevation of NGAL suggests ongoing kidney damage, which implicates the inflammatory cascade and releases calprotectin.^56^ Second, our risk-evaluation strategy might have excluded patients who exhibited confounding factors that interference biomarkers. Thus, single biomarker to detect intrinsic AKI was recommended. This might also explain that calprotectin, rather than NGAL, was the only biomarker retained in the multivariate analysis for intrinsic AKI.

Currently, AKI definition was based on Cr change, this might decrease the diagnosis of AKI, since some patients already had AKI at the time of admission. On the other hand, these patients could have higher levels of biomarkers, interfering with their performance to predict AKI and its associated prognosis. We acknowledge Chen et al^8^ for this work. Thus, NGAL and calprotectin have its clinical application for intrinsic AKI. Furthermore, to insure the influence of urine concentration on biomarkers, the levels of NGAL and calprotectin were adjusted to urine Cr (Figure 1). In view of baseline differences and dynamic change of Cr excretion during AKI, it may be useful to compare the normalized biomarker levels to baseline, in order to account for inter-individual differences in baseline Cr excretion rates.^57^ This area deserves further investigation. The possible explanation large variability within group is that the level of these markers might reflect the severity of disease and also predict the prognosis. The appendices 3 and 4, http://links.lww.com/MD/A447 illustrated the higher mortality rate in patients with NGAL exceeding39.0 ng/mL or calprotectin exceeding 314.6 ng/mL.

This study has several limitations. First, only 1 measurement of the NGAL and calprotectin levels was used in this cross-sectional study to predict the incidence of intrinsic AKI. Repeat measurements to detect secondary kidney damage may improve the predictive ability. Second, the roles and expression of calprotectin in chronic active glomerulus nephritis require further investigation. Third, we excluded urinary tract infections and gross hematuria related to catheter trauma that influences the urinary NGAL and calprotectin levels. Therefore, meticulous clinical inspection and interpretation methods must be implemented for asymptomatic pyuria. Fourth, this study considered AKI identified in a 7-day period alone; thus, studies with long-term follow-up periods are warranted to explore the relationship between mortality and the concerned biomarkers. Fifth, this research was conducted on a heterogeneous population, and no subgroup analysis was conducted to explore the relationships between a specific disease type and the biomarkers. Finally, considering the small sample size and observational design, additional prospective randomized trials are warranted for verifying the cost efficacy of using these markers to modify clinical pathways. The assessments of NGAL and calprotectin levels were expensive and time consuming. There are few data on the influence of prolonged duration of storage at −80°C on the stability of urinary markers at the present time.^55,58^ Before clinical application, prospective studies including healthy individuals should be performed to standardize the protocols and facilitate the creation of reproducible assays.

In summary, we present 2 inferences of this investigation. First, an appropriate risk-evaluation strategy could improve the detection rate of AKI and reduce the cost of biomarker assessments in clinical practice. Second, increased urinary NGAL and calprotectin levels are both accurate in distinguishing intrinsic AKI from prerenal azotemia in a CCU setting. The novelty of present study could be a reliable noninvasive test in clinical implication to differentiate intrinsic from prerenal AKI and it would shorten the time to initiation of appropriate therapy. Increased urinary calprotectin levels serve as independent and accurate predictors of intrinsic AKI. The results indicate the crucial role of inflammation as an unfavorable factor in persistent kidney damage. Accordingly, careful inspection for medication, choice of therapy, and early intervention in patients exhibiting increased biomarker levels might improve the outcomes of kidney injury.
